# Converging theories on dreaming: Between Freud, predictive processing, and psychedelic research

**DOI:** 10.3389/fnhum.2023.1080177

**Published:** 2023-02-16

**Authors:** Michael Koslowski, Max-Pelgrom de Haas, Tamara Fischmann

**Affiliations:** ^1^Department of Psychiatry and Psychotherapy, Charité CCM—Corporate Member of Freie Universität Berlin, Humboldt-Universität zu Berlin, and Berlin Institute of Health, Berlin, Germany; ^2^Clinical Psychology and Psychoanalysis, International Psychoanalytic University Berlin, Berlin, Germany; ^3^Focus III: Psychotherapy and Psychoanalytic Conceptual Research, Sigmund-Freud-Institut, Frankfurt am Main, Germany

**Keywords:** dreaming, predictive processing, prior, dream coding, psychedelics, psychoanalytical, consciousness

## Abstract

Dreams are still an enigma of human cognition, studied extensively in psychoanalysis and neuroscience. According to the Freudian dream theory and Solms' modifications of the unconscious derived from it, the fundamental task of meeting our emotional needs is guided by the principle of homeostasis. Our innate value system generates conscious feelings of pleasure and unpleasure, resulting in the behavior of approaching or withdrawing from the world of objects. Based on these experiences, a hierarchical generative model of predictions (*priors*) about the world is constantly created and modified, with the aim to optimize the meeting of our needs by reducing prediction error, as described in the *predictive processing* model of cognition. Growing evidence from neuroimaging supports this theory. The same hierarchical functioning of the brain is in place during sleep and dreaming, with some important modifications like a lack of sensual and motor perception and action. Another characteristic of dreaming is the predominance of *primary process thinking*, an associative, non-rational cognitive style, which can be found in similar altered states of consciousness like the effect of psychedelics. Mental events that do not successfully fulfill an emotional need will cause a prediction error, leading to conscious attention and adaptation of the priors that incorrectly predicted the event. However, this is not the case for *repressed priors* (RPs), which are defined by the inability to become reconsolidated or removed, despite ongoing error signal production. We hypothesize that Solms' RPs correspond with the *conflictual complexes*, as described by Moser in his dream formation theory. Thus, in dreams and dream-like states, these unconscious RPs might become accessible in symbolic and non-declarative forms that the subject is able to *feel* and make sense of. Finally, we present the similarities between dreaming and the psychedelic state. Insights from psychedelic research could be used to inform dream research and related therapeutic interventions, and vice versa. We propose further empirical research questions and methods and finally present our ongoing trial “Biological Functions of Dreaming” to test the hypothesis that dreaming predicts intact sleep architecture and memory consolidation, *via* a lesion model with stroke patients who lost the ability to dream.

## 1. Introduction: Freud, homeostasis, feelings, and predictions about the world

When Freud stated in 1894 that “quotas of affect spread over the memory-traces of ideas somewhat as an electric charge is spread over the surface of a body” (Freud, 1894), he laid the ground for what is now common knowledge in affective neuroscience that arousal processes arise in the brainstem and are felt as affects which are distinct from memory-traces of ideas (Solms, 2013). The latter are representational processes that involve forebrain and cortical processes. According to Freud, the distinction between representational processes and *quotas of affects* that are activated by these arousals lays the ground for how the brain works, namely, by feelings. Consciousness registers the state of the subject by feelings and not that of the object world, namely, perception. Freud went on to consider: “If now we apply ourselves to considering mental life from a biological point of view, an instinct appears to us as a concept on the frontier between the mental and the somatic, as the psychical representative of the stimuli originating from within the organism and reaching the mind, as a measure of the demand made upon the mind for work in consequence of its connection with the body” (Freud, 1915). This points to how we understand brain processes that give rise to pleasant and unpleasant affects in relation to the mechanism of homeostasis.

Freud thought that we must turn to biology because instincts [Triebe] are fundamentally biological processes. According to his theories, the fundamental developmental task is to learn how to meet our needs in the world and how to manage our emotional needs. The homeostasis principle can guide us in this regard by introducing a value system giving direction if we approach or withdraw from the world of objects, to maintain a state of homeostasis where our needs are met (Solms, 2019). This value system is pleasure and *unpleasure* (emotional pain) as affective signals that are felt when our needs are met or not. When Freud realized from his clinical observation that humans are not only searching for pleasure but are also looking for something “deeper,” beyond pleasure, he called it “the Nirvana principle” (Freud, 1920): no needs, no demands upon the mind, which he distinguished from the pleasure principle. But now, we know that they are not two separate principles; the pleasure principle is *in the service of* the Nirvana principle. They are one and the same principle called the homeostasis principle. The state of Nirvana conceptualized from the perspective of the principle of homeostasis is the state of no need, which can biologically be seen as the “ideal life situation” (Carhart-Harris and Friston, 2010; Solms, 2018). Whether our prediction of how to meet our needs is accurate or not, this is the work the mind has to perform in the service of homeostasis to stay within our viable bounds. *Feelings* can be described as the mental and affective representation of how we maintain homeostasis. We are born with certain predictions of how to meet our needs, and those innate predictions are called reflexes and instincts. Reflexes and instincts yet seem to lack the complexity to meet all human needs. They are automatic, stereotyped responses, while humans need to behave and react in different, adaptive ways depending on the situation in question. We are required to learn from experience how to satisfy our needs. This means we must supplement our innate predictions as they are too crude. We need more context-related methods to satisfy our drives. That is the whole task of ego-development, of learning from experience, and of building an internalized representation of how the world works because it is in the world where we have to satisfy our drives. The psychoanalytic *Ego* is all about predictions. Its task is to learn from the experience of the past to predict how to go about meeting our needs in the future (Carhart-Harris and Friston, 2010; Solms, 2018). To the extent that we learn predictions that are not adequate and do not work, to that extent we suffer from feelings.

## 2. Predictive processing and the dreaming brain

Dreaming always had a strong significance in Freud's core concepts of human consciousness, the ego, and the unconsciousness; he considered dreams the “royal road to the unconscious” (Freud, 1900). Many of Freud's initial theories have been successfully updated and aligned with modern psychological and neurobiological frameworks (Solms, 2018). One currently influential model within computational neuroscience and epistemology is the predictive processing (PP) account. We will demonstrate in the following analysis and comparison of different theories and aspects from different perspectives that the PP model can reasonably be applied to a psychoanalytical understanding of dreaming and that promising insights and new hypotheses can be derived from this cross-theoretical approach.

The PP or predictive coding theory of brain functioning has its roots in Bayesian probability statistics. Bayesian statistics describe the uncertainty of events in terms of their mathematical probability, meaning the degree of belief in an event based on up to that point acquired knowledge (Spiegelhalter and Rice, 2009). The PP model offers a unifying framework for imagination, perception, and the brain's organization as a sense-making-organ, which tries to find causation within the world that surrounds it (Clark, 2012; Hohwy, 2013). It postulates that the brain can be understood as a hierarchically structured inference system, operating under the premise of minimizing long-term prediction error. Within these multilayered structures, each layer generates expectations, the so-called priors, of what sensory information it will receive by the next layer below. Depending on the layer's hierarchical position, these priors vary in their degree of abstraction and temporal reference. Bottom layer predictions deal with detail-rich, fast-scale perceptual elements, getting progressively oriented toward long-term meta-features at the top. Predictions are then matched with the actual bottom layer input, resulting in a quantifiable prediction error depending on the discrepancy between input and expectations. Over time, the system will enhance the accuracy of its priors to make an adequate assumption about internal and external conditions. Depending on the environment and the task to be accomplished, the weighting of prediction error signals can be dynamically adjusted. For example, in environments in which sensory signals are weak or highly fault-prone (“noisy” conditions, like visual perception in darkness), the dependency on higher-level priors is increased, while lower-level priors are attenuated. PP's synthesis of active “world-creation” and passive “world-sensing” enables an integrated understanding of mental operations previously conceptualized rather separately. As Clark expressed: “Perceivers like us … are inevitable potential dreamers and imaginers too” (Clark, 2012).

Since the PP model gained momentum in cognitive neuroscience, there have been attempts to apply the PP account not only to waking consciousness but also to dream. Clark, Hobson, and Friston argue that the same hierarchical functioning is in place during sleep, with the exception of a lack of sensitive and motor perception and action (Crick and Mitchison, 1983; Hobson and Friston, 2012; Hobson, 2014; Clark, 2016). For a more detailed review of the application of the PP account on sleep and dreaming, refer to the study by Bucci and Grasso (2017). According to the authors, REM sleep forces the brain to exclusively rely on its middle-to-high-level priors to deal with random neuronal activity since low-level priors are evaluated as unreliable. This explains the dreamscape's lack of “the fine-grained perceptual details and depth” compared to waking life (Bucci and Grasso, 2017). While this explanation relies predominantly on the neural signature of REM sleep, there is growing evidence that dreaming also happens during NREM sleep and that there might be continuity between different states of consciousness (e.g., waking, daydreaming, and dreaming) involving complex cortical activation patterns (i.e., the default mode network [DMN]), which does not contest the basic PP hypothesis for dreaming (Domhoff, 2011; Fox et al., 2013).

## 3. From Freud to Friston: Primary process and free energy principle

The distinction between *primary and secondary processes* is a classic idea of Freudian psychoanalysis (Freud, 1900). It accounts for the fundamentally different modes of cognition between ordinary, adult waking consciousness, and non-ordinary states such as dreaming, psychosis, or infantility. While the former is characterized by an ordered, rational, and coherent style of mental operations, impulse control, and reality testing, the latter is described as the persistence of more primitive and chaotic forms of thinking, essentially being a regressive, developmentally outgrown type of *protoconsciousness*. According to Freud, the primary process describes the cognitive style of the *id/unconscious*, and the secondary process is the one of the *ego* or, in less psychoanalytical terms, the cognitive functioning of a healthy, rational human mind in its waking state (Freud, 1940). In this framework, dreams are generated in the pre-verbal, unconscious space of the primary process. For a more thorough, critical discussion of the concept of the primary process and its relation to primordial consciousness and repression, refer to the study by Robbins (2018).

In the states of sleep and dreaming, the same dopaminergic SEEKING system (Panksepp and Wilson, 2016; capital letters by the authors) is active as in waking life. However, in sleep, the sensory input is reduced to a minimum. This makes the brain free to minimize complexity in REM dreaming and hence assumedly resolves otherwise disruptive strong emotional reactions, which could interrupt continuous sleep. In psychoanalytic dream theory, it is assumed that impingements from inside (emotional needs) or outside (life events and day residues) are major causes and sources for dreaming (Freud, 1900).

This aspect has found access to Friston's concept of free energy (FE) (Friston, 2012). Friston hypothesizes that the brain operates to minimize FE caused by sensory impingements of unpredicted stimuli. Similar to Freud who assumed major needs or biological imperatives to reflect such impingements, Friston proposes that these sensory impingements reflect compliance with biological imperatives, creating a “demand for work” (Freud, 1915) to produce “specific actions” or, to put it in Friston's words, “an imperative to minimize prediction error … through action” (Friston, 2012). For both Freud and Friston, when such sensory impingements are felt, they put a demand on our brain to embody a representation of these sensory impingements including representations of the bodily ego (Freud, 1923) or as Friston would term it the “agent's body.” These bodily representations are initially met by an innate generation of a prior virtual version of reality, i.e., a constructed and simplified version of reality, which will subsequently be modified by experience.

We might say from here that Freud's primary process and Friston's “virtual reality generator” can be seen as innate producers of imaginary prior beliefs and predictions of the actual experience. According to Freud, the primary process is “in the apparatus first,” which could be termed in the PP model as higher-order priors or beliefs that are usually not accessible to consciousness, hence unconsciousness (Freud, 1900).

According to other influential dream theories (threat simulation theory and social simulation theory), dreaming can be described as a perceptual synthesis by testing real-life experiences in a virtual setting, co-creating, and updating a generalizable generative model of the world, in order to simulate threatening or important social situations (Revonsuo, 2000; Ruby, 2011; Tuominen et al., 2019; Scarpelli et al., 2022). The generative model is thereafter tested in conscious waking life for its feasibility and precision of the prior predictions, producing prediction errors and, thus, “surprise,” change in behavior, and further model updating, when real-life perceptions differ from the predictions.

In conclusion, the PP account is well compatible with both traditional psychoanalytical dream theory and more recent cognitive dream theories.

## 4. The repressed prior

Mark Solms' core conception called “predictive processing and the feeling brain” is a Neo-Freudian, neurobiologically informed model based on the principle of the homeostasis of feelings, which is governed by the pleasure principle (Solms, 2018, 2021). For our focus on dream research, the unconscious is of particular interest. In Solms' conception, the unconscious consists of different functional subsystems, which can be mapped onto distinct brain networks:

1. The “system unconscious,” where “the repressed” is derived from cognitive (representational) processes, acquired by non-declarative learning. Its functions are performed by subcortical brain structures (basal ganglia, cerebellum).
  - Cognitive unconscious: *legitimately* (maturely) automatized predictions; the normal case, because “predictions work well”.
  - Dynamic unconscious: *illegitimately* (prematurely) automatized predictions, the repressed; the pathological case, because the need could not be satisfied (e.g., repressions due to the Oedipus complex) → *the repressed prior* (RP).
2. The “preconscious,” consolidated declarative memory content, is performed by the cortex.

Following this model, mental events that do not successfully fulfill an emotional need cause a prediction error, leading to conscious attention, problem-solving, consecutive reconsolidation and adaptation of the priors that predicted the event. However, this is not the case for *repressed priors*, which are defined by the inability to become reconsolidated or removed, despite ongoing error signal production (Solms, 2018). The RP is described as a prematurely formed, maladaptive automatization of the infantile prediction of an apparently insolvable problem (conflict). There is no explicit mental representation of an RP that could be experienced or verbalized, and its impact remains in the unconscious, affective layers of consciousness. This automatization might follow the economic rationale that a better solution cannot be found at the current moment and a conscious re-engagement with the problem would occupy too much of the capacities of working memory, thus preventing it from dealing with issues it can actually solve (Solms, 2018).

Interestingly, the concept of the RP shares some core aspects with the *unsolved conflictual complexes*, as described by Moser (Moser and von Zeppelin, 1996; Moser and Hortig, 2019), which is presented in the following section. By doing so, we intend to bridge a gap between the PP account of dreaming and the psychoanalytically inspired dream generation model of Moser et al.

## 5. The psychodynamic dream generation model

Moser's dream generation model (Moser and von Zeppelin, 1996; Moser and Hortig, 2019) is based on psychodynamic dream theory, developmental and cognitive psychology, as well as experimental dream research. Moser et al. consider the sleep dream as a simulated *micro-world* controlled by affectivity, which generates images of entities involved in it and their relations to each other. A dream is triggered by current concerns of events that happen during the day (day-residues), which are capable of reactivating unresolved conflicts and problems due to structural similarities. The dream having the function to resolve those conflicts can do so more readily in contrast to the waking state, as the dream state has no capacity restrictions of the memory system. Consolidation processes can also take place during sleep in the so-called *off-line mode*. This is how new information is integrated into long-term memory while sleeping. The range of affect modulation is significantly larger in the micro-worlds of dreaming and stress is absorbed readily both *via* imagination and cognition. The dream is not involved in regulating concrete-real object relationships but rather works with memories, acquired solutions, and defense strategies, which are regrouped as *prototypical affective microprocesses* (PAMs).

A dream, which is usually pictorial, consists of at least one situation produced by a *dream-organizer*. According to Moser, a dream-organization may be considered a bundle of affective-cognitive procedures (i.e., PAMs), generating the micro-world dream and controlling its course of action. Within this system, the so-called *dream-complex* serves as a template for facilitating dream organization. Thus, it may be assumed that a dream-complex originates from one or more complexes stored in long-term memory, rooted in conflictual or traumatizing experiences, which found their condensates in introjects. These conflictual or traumatic dream complexes are easily triggered by stimuli from the outside world, which are structurally similar to stored situations of these complexes. Searching for a solution for this complex is governed by the need for security and wish for involvement, i.e., the *security-principle* and the *involvement-principle* which govern dream-organization.

Within these complexes, wishes are the links between PAMs of self and objects, which are accompanied by cognitive aspects such as convictions and hope for wish-fulfillment. *Conflictual complexes* are areas of PAMs with a repetitive character, thus creating areas of unbound affective information. Affects within such an area are inter-connected but blocked from memory and, thus, not accessible for our conscious cognition. They are the integrated affects, which due to their lack of representational contextualization can hardly be modulated or resolved, and that makes the patient suffer from conflictual complexes. To solve these conflictual or traumatic complexes, it is necessary to retrieve the affective information and re-integrate them into a relational reality to make the complex “come alive.” This is being attempted in dreams whose function is to search for a solution to the complex. The search for a solution within a dream is governed by the abovementioned need for security and a wish for involvement.

In order to collect empirical data about the dream generation described in Moser's theoretical framework, a standardized method to codify the manifest dream was developed, the *Zurich Dream Process Coding System* (ZDPCS). It enables to scientifically measure alterations in dream contents, which has been shown in several empirical studies (Fischmann et al., 2013, 2021; Fischmann and Leuzinger-Bohleber, 2017; Wittmann et al., 2017).

We will later propose that the use of instruments like the ZDPCS would be an interesting tool to investigate also other, dream-like states of waking consciousness, e.g., the psychedelic experience, which is the state of the brain and consciousness during the influence of a serotonergic (5HT-2a receptor agonist) substance. Those states can be used as a promising experimental condition to better understand the process and structure of dream generation, from both a phenomenological-psychological and a neurobiological perspective. On the neurobiological level, PP could be the best available framework to link these phenomenological data with cognitive neuroscience methods and paradigms.

## 6. Repressed priors and conflictual complexes: Bridging the two models

We discussed earlier that RPs, according to Solms, are automated response patterns of the non-declarative long-term memory system. They constantly produce prediction error signals, which can be “felt” as unpleasure, but they cannot be “thought” as they lack properties of cognitive representation. In psychodynamic terms, they are repressed to the unconscious. The RPs are described as not having a representable form and, thus, could not become reconsolidated whatsoever. We hypothesize that Solms' RP corresponds to the conflictual complex (CC) in Moser's theory of dream generation, i.e., the RP/CC is considered one single entity, described from two different angles. See Table 1 for a comparison of the core features of RP and CC. We agree though that an RP/CC could possibly never be expressed *directly* in a cognitive representation—one needs to dream, or enter a dream-like state, to make the unbound affect (according to Moser)/unfulfilled emotional need behind the error accumulation (according to Solms) accessible to further processing in a represented manner.

**Table 1:** Repressed prior and conflictual complex—comparison of core features.

	**Repressed prior (Solms)**	**Conflictual complex (Moser)**
Features	Unconscious	Blocked from consciousness
	No cognitive representation	Association to current concern elicits dream
	Automatized response	Unbound affective information
Origin	Repressed due to unsolvable conflict	Repressed due to unresolved conflict
	Acquired in early Development	
Dynamics	A priori non-representable (only the affective part)	Associated to current concern making it accessible
		Access possible (e.g., by dream work in psychotherapy)

Thus, we propose that in line with Moser's dream theory and Solms' neuropsychodynamic framework, the underlying emotional need, which these priors attempt but fail to satisfy, can become contextualized and experienceable *within dreams and similar states of experience and neurobiological functioning*. Dreaming might be a state of cognition in which these unresolved emotional needs can be approached with alternative solutions for wish-fulfillment and conscious access. In the PP model, this would mean that the affective consequence of the malfunctioning RP shapes the conscious experience of the dream, e.g., in the form of an affect-laden symbol, with semantic associations between dream content and self-narratives. The following processing of the transformed content during the waking state, e.g., generating personal meaning of a bizarre dream like one does in psychoanalytical dream interpretation, might then result in new insights about maladaptive (pathological) patterns of prediction of inner and outer world events, and potentially reduce cognitive, affective, and behavioral rigidity. This dynamic process might facilitate new predictions and shape behavioral reactions that are more capable of meeting unmet emotional needs. The dream state might thus continue and potentiate processes that might have been initiated previously during waking consciousness. To account for the proposed function of dreams, they need to be understood as presenting a categorically different “mode of operating” or “style of cognition” than that exhibited by the brain during ordinary waking consciousness—primary process thinking, as opposed to secondary process thinking, as we stated earlier.

We hope that by understanding dreaming as a primary process, we can build a hypothesis to account for two things. First, dreams can incorporate the conflicted complex in ways waking consciousness cannot. Second, how the dream state might facilitate alternative ways of solving these complexes (unfulfilled emotional needs) to settle in.

We hypothesize that with the weakening of ego-control and its secondary defenses, the prediction error of the repressed prior can become felt as the unmet emotional demand it fails to fulfill. On a neurobiological level, one possible explanation might be that as the default-mode network's (DMN)^1^ control over the medial temporal lobe (MTL)^2^ decreases within primary consciousness (DMN-MTL decoupling, Carhart-Harris et al., 2014), affective impulses might then activate associatively connected contents of the declarative memory systems in a bottom-up manner and become integrated *via* the association cortices around the TPO-region (Solms, 2000) into the embodied, simulated micro-world of the dream state.

We hypothesize that with the strong restriction of external sensory input and the decreased precision weighting of high-level priors, the generative freedom for action and scenarios beyond the rules of everyday reality and entrenched reaction patterns substantially increases. A significant function of high-level priors is the stratification of inferior levels within the inferential hierarchy, channeling or streamlining predictive roads^3^, which are the most in line with the core assumptions of the system. The result is a constrained amount of prioritized, pre-structured predictive paths (the metaphor of predictive highways might be suitable) in accordance with Occam's principle, which enables efficient functioning (control and agency) during waking activity. These earlier prioritizations may either be legitimate, meaning that they have proven to work well in fulfilling the need they serve and have been, therefore, correctly automatized (Solms' cognitive unconscious) or illegitimate in the context of what has been called the dynamic or repressed unconscious (Solms, 2018). Again, in this terminology, repression means that this prediction remains protected from reconciliation (*via* precision weighting) despite its production of error signals.^4^

With the reduced weighting of higher-level priors, their restrictive control on lower levels diminishes and gives rise to less pre-emphasized and combinatory unrestrained mid-level priors. Sticking to our metaphor, our highways have turned into normal roads and resemble just one option besides others which are now more accessible. This in turn, as stated previously, might facilitate newly learned, more mature predictions to be tried out in the “safe space” of the dream-world and continue to settle in.

## 7. Neural dynamics of the primary process: Data from research with psychedelics

Recently, with the increase in neuroscientific research on psychedelic states, the concept of primary and secondary process thinking has regained academic interest and popularity. A pioneering study by Carhart-Harris et al. aligned these conceptions with the predictive brain theory and the entropic brain hypothesis (Carhart-Harris and Friston, 2010; Carhart-Harris et al., 2014), arguing that the essential quality of the secondary process is to minimize free-energy (entropy, uncertainty) *via* top-down predictions which suppress occurring prediction errors on lower levels of the inferential hierarchy. They provide empirical data that imply that psychedelics disrupt these processes, particularly neural activity in the DMN, and induce a primary state of consciousness that is hypothesized to have specific underlying neurophysiological characteristics. This disruption of the hierarchical predictive architecture and a compromised capacity of top-down control results in less constrained cognitions and more chaotic (higher-entropy) neural dynamics, which on the subjective level goes along with primary process thinking. Summarized in the REBUS formulation (“relaxed beliefs under psychedelics”), the authors argue that in these states, high-level priors become deemphasized *via* reduced *precision-weighting* and allow for a broader range of lower-level activity to occur, explaining the distinct phenomenology of the psychedelic experience (Carhart-Harris and Friston, 2019). Other research groups stressed different aspects of brain activation patterns, e.g., dysfunction of thalamic gating, leading to very similar conclusions for subjective experience and brain functioning in the psychedelic state (Vollenweider and Kometer, 2010; Preller and Vollenweider, 2018).

## 8. Dreaming and the psychedelic experience

It has been stated repeatedly that the phenomenology of dreaming shares many similarities with the subjective experience after ingestion of a psychedelic substance with strong agonism at the serotonin (5-HT)-2A receptor, such as psilocybin, LSD, mescaline, or DMT/Ayahuasca (Schultes and Hofmann, 1979; Kraehenmann, 2017; Palhano-Fontes et al., 2021). A study comparing dream reports and reports of psychedelic experience found perceptual changes and close relationships as the most prevalent themes in both conditions (Sanz et al., 2018). The phenomenology of the psychedelic experience has been assessed and mapped by the use of several standardized questionnaires and qualitative interviews in relation to the dose of the applied psychedelic substances (Griffiths et al., 2006; Studerus et al., 2011; Millière, 2017; Preller and Vollenweider, 2018; Holze et al., 2021). The available quantitative data in relation to the applied dose of psychedelics have recently been regrouped in the Altered States Database project (Schmidt and Berkemeyer, 2018).

In a study with healthy subjects performing a mental imagery task after the ingestion of a high dose of the psychedelic LSD, the authors observed a shift of subjective experience toward the abovementioned primary process thinking (Kraehenmann et al., 2017). The use of the term “primary process thinking” here follows the conceptualization of Auld (Auld et al., 1968), who developed a scale for the evaluation of dream reports. This scale sums up scores in nine categories, namely, condensation, unlikely combinations or events, fluid transformations, visual representation, symbolism, contradiction, magical occurrences, inhibited movement, and taboo sexual and aggressive acts. These elements were then related to secondary process thinking, to acquire the *primary index* (PI), as established in studies on guided mental imagery and daydreaming (Stigler and Pokorny, 2001). The authors concluded that both dreaming and the psychedelic state share a distinct mode of cognition characterized by primary process thinking.

Concerning the experiential domain of the sense of self in altered states of consciousness such as sleep, dreaming, meditative states, and the psychedelic experience, there is a growing body of literature bridging core concepts from the philosophy of mind and cognitive science (Letheby and Gerrans, 2017; Millière et al., 2018). In the case of the psychedelic experience as a “lab model” for the study of altered states of consciousness, there is convincing empirical evidence in favor of a stepwise disintegration in the sense of a coherent, enduring, temporally and spatially well-defined self-as-object. This process can be seen as *self-unbinding* (Letheby and Gerrans, 2017), extending the model of cognitive binding (Sui and Humphreys, 2015) to explain the psychedelic-induced loss of ego functions and body boundaries. This model is simultaneously informed by the abovementioned account of PP and the REBUS model, allowing empirical hypothesis testing with neuroimaging methods. This seems a fruitful direction to further investigate changes in self-experience in dream research.

Comparing the phenomenology of dreaming and the experiences induced by high doses of a psychedelic^5^, the following commonalities and differences can be described.

The similarities in phenomenology between dreaming and the psychedelic experience are obvious and manyfold, also with more fine-grained distinctions of the different features (Edwards et al., 2013; Kraehenmann, 2017; Kraehenmann et al., 2017; Letheby and Gerrans, 2017; Schmidt and Berkemeyer, 2018). Table 2 shows strong similarities in the domains of perception, affect, and cognition.

**Table 2:** Phenomenology of dreaming and the psychedelic experience.

	**Domains of phenomenology**				
	**Perception**	**Affectivity**	**Cognition**	**Sense of self**	**Behavior**
Shared phenomenological features: Dreaming and psychedelic experience	Vivid, predominantly visual imaginary perceptive changes; bizarreness; symbol formation	Strong activation of emotional memories and affects (positive and negative valence); retrieval of fear memory: nightmare/“challenging experience”, “bad trip”	Decrease of logical and increase of associative reasoning; shift toward bizarre, symbolic and metaphoric thinking; insightfulness; primary process thinking	Disintegration of narrative and embodied (minimal) self, non-dual awareness, depersonalization/ derealization	
**Differences in phenomenology**					
Dreaming	REM dreams: mostly complex images; influence of external stimuli is marginal (“slamming door”)		No meta-cognition/reality monitoring; memory functions partly preserved; exception: lucid dreams		Lack of motor control; exception: lucid dreams (voluntary control of eye movements)
Psychedelic experience	Complex images and elementary percepts (abstract geometrical forms) mental imagery modified by external perception (e.g, synesthesia)		Metacognition/reality monitoring and memory functions mostly preserved		Motor control mostly preserved

Regarding the differences, we are inclined to locate the psychedelic state closer to lucid dreams (LD) than regular (REM-) dreams, as in LD meta-cognition, reality monitoring and memory functions are preserved, conscious choices can be made, and real-time communication is possible *via* eye movements (Dresler et al., 2014; Baird et al., 2019; Mota-Rolim, 2020; Loo and Cheng, 2022). Real-time communication offers new possibilities for empirical research and improves the precision and richness of subjective reports, as research on regular dreams almost always suffers from the indirect, *a posteriori* nature of dream reports.

The specific feature of elementary visual imagery in the psychedelic state (e.g., circles, triangles, colored patterns on object surfaces, halo effects) might be due to the neurobiological feature of the 5HT-2A-receptor agonism of psychedelics, inducing changed sensory drive and temporal dynamics in the visual cortex (V1) which in turn lead to impaired integration of visual perception (Michaiel et al., 2019). This difference might limit the comparability of the two states.

In dreams, there is an obvious lack of external stimulation, whereas, in the psychedelic state, external stimuli are known to have a substantial influence on the subjective experience. This difference might play a minor role in current empirical research, as in the standardized experimental setting of modern clinical trials with psychedelics, the subjects wear eyeshades, have their eyes closed, and are asked to direct their attention toward the “inner world,” focusing on arising images, feelings, thoughts, and somatic experiences—the “outer world” stays mostly outside (Koslowski et al., 2021). Still, the fact that many trials include listening to emotionally activating music might be a source of bias in the direct comparison of these states.

The lack of motor control in dreams seems to be another important difference, from the perspective of embodied mind regarding motor-proprioceptive feedback loops, while we should bear in mind that brainstem atonia in REM sleep is not as complete as one might think (Windt, 2015).

Taken together, we think that the phenomenological similarities between dreaming and the psychedelic state outweigh by far their differences. Particularly, there are many structural similarities from the perspective of the visual, affective, and cognitive features.

## 9. Discussion

In the sections 1 to 6, we discussed how the different features of dreaming can be reasonably described in terms of the PP model of cognition and that a current theory of dream generation (Moser) is well compatible with this view. We argued for the commonalities of Solms' repressed prior and Moser's conflictual complex, which could help inform both theories with testable hypotheses. In section 7, we introduced the neurocognitive perspective on the psychedelic experience, which, like dreaming, has been explained in terms of the PP model. In section 8, we reviewed the phenomenological similarities and differences between dreaming and the psychedelic experience and proposed the application of a psychedelic substance as an experimental model to induce a dream-like state in waking consciousness, which would allow us to expand the limits of dream research due to improved perception and communication of the actual experience.

Following the arguments in the sections earlier, we derive the following propositions for future dream research:

1. The earlier mentioned dream coding system ZDPCS could be used in empirical trials to further investigate the described similarities in the formal structure of experience in dreaming, normal waking state, and dream-like waking states, e.g., pharmacologically induced by the application of psychedelic substances.
2. Insights from psychodynamic dream work could be therapeutically relevant for psychedelic-assisted therapy, as there is still an ongoing discussion on which psychotherapeutic model one should apply (Wolff et al., 2020; Koslowski et al., 2021; Yaden et al., 2022). The repressed priors/conflictual complexes of the PP account of dreaming could possibly be “traced,” reconstructed, and reconciled, at least their affective part, using specific psychodynamic interventions (i.e., free association, symbolization, and psychodynamic interpretation of the experienced content).
3. Functional neuroimaging paradigms and other neuroscience experiments from research on psychedelics could be used in different sleep stages (REM/non-REM) and dream conditions (regular dreaming/lucid dreaming) to further elucidate the mechanisms of dream formation. This is particularly interesting for paradigm testing for the different layers of the PP account (e.g., visual system, emotion processing, higher-order beliefs, and sense of self).
4. Lesion models that mimic the loss of the ability to dream could shed light on the proposed biological and evolutionary functions of dreaming, as stated by Freud and Solms. One could examine patients with a distinct brain lesion associated with the ability to dream to further investigate some of the above-described hypotheses.

The fourth proposal in this list, brain lesion models for dream research, is about to be realized in our ongoing observational trial “BFD—Biological Functions of Dreaming” (https://clinicaltrials.gov/ct2/show/NCT04749992). This project aims to understand the biological function of dreams, which differs from that of REM sleep. Based on Mark Solms' neuropsychoanalytical theory and neuropsychological findings that REM sleep and dreams are doubly dissociable phenomena (Solms, 2000, 2014), Freud's central hypothesis that dreams serve to maintain sleep will be investigated further (Freud, 1900). By this, Freud meant that the dream is a response to affect-laden impulses for action with hallucinatory wish-fulfillment so that it does not lead to awakening. Second, it will be examined whether dreams influence the consolidation of affective and non-declarative (motor) memory. Our hypotheses are that patients who have lost the ability to dream during REM sleep have poorer sleep quality and poorer emotional and non-declarative memory consolidation. This will be investigated in two groups of neurological patients with thrombotic infarction in the area of the posterior cerebral artery (PCA) who have lost the ability to dream while retaining REM sleep. The PCA stroke was selected as a lesion model because it has been shown that a lesion in the temporoparietal junction and related structures in the PCA area frequently led to a loss of dreaming (Solms, 2014).

We hope that our considerations might inspire other researchers to take further steps in the interdisciplinary terrain of empirical research on dreaming and altered states of consciousness.

## Author contributions

MK and M-PH conceived the central theoretical ideas presented in this article. TF contributed to Mosers theory of dreaming and provided critical feedback. MK, M-PH, and TF had equal contributions and wrote the manuscript. All authors contributed to the article and approved the submitted version.

## Footnotes

^1^According to Carhart-Harris and Friston (2010), the DMN represents the neurological correlate of the psychoanalytic ego.

^2^The MTL including structures such as the hippocampus, amygdala, and para-hippocampal regions plays a central role in the functioning of the declarative memory system (Cutsuridis and Yoshida, 2017). Damage of these structures does not cause secessions of the ability to dream all together (Solms, 2000), but does impair the expressiveness of dream reports. For example, bilateral hippocampal lesions have been reported to reduce the episodic quality and content-richness of dreams (Spanò et al., 2020), and dreams of patients with bilateral damage of the amygdala appeared to be significantly shorter and less complex (Blake et al., 2019).

^3^We chose the term “predictive roads” to illustrate that the deeply consolidated priors at the core of the predictive hierarchy (non-declarative LTM priors in form of “action tendencies”) channel or orchestrate the consecutively following predictions on inferior levels such as motor-programs, mental images, declarative contents, and so on. This is how our meta-priors choose or direct the downstream routes our predictive cascades may take.

^4^Importantly, Solms proposed the conception of repression with regard to unsolvable problems argues along a similar mechanism as Carhart-Harris' proposition for adverse life events (Carhart-Harris, 2019): ‘The mnemonic Tightened Beliefs in Response to uncertainty (TIBER) is offered to capture this phenomenon. Here “trauma” is interchangeable with “uncertainty”—if used in an extended sense to mean any significant acute and/or repeated adversity that is paralleled by significant uncertainty. The basic tenet is that (implicit) beliefs tighten as a defensive response to significant, intolerable stress and uncertainty.” Insofar, one might suspect that Moser's ‘conflicted, traumatic complex' also seem to root in a consistent underlying mechanism of aberrant precision-weighting.

^5^Most of the recent clinical trials use Psilocybin 25 mg or LSD 100–150 μg as a single high dose to induce the full spectrum of a psychedelic experience.

## References

[B1] Auld F. Goldenberg G. M. Weiss J. V. (1968). Measurement of primary-process thinking in dream reports. J. Pers. Soc. Psychol. 8, 418. 10.1037/h0025488 4171430

[B2] Baird B. Mota-Rolim S. A. Dresler M. (2019). The cognitive neuroscience of lucid dreaming. Neurosci. Biobehav. Rev. 100, 305–323. 10.1016/j.neubiorev.2019.03.008 30880167 PMC6451677

[B3] Blake Y. Terburg D. Balchin R. van Honk J. Solms M. (2019). The role of the basolateral amygdala in dreaming. Cortex. 113, 169–183. 10.1016/j.cortex.2018.12.016 30660955

[B4] Bucci A. Grasso M. (2017). Sleep and Dreaming in the Predictive Processing Framework.

[B5] Carhart-Harris R. (2019). How do psychedelics work? Curr. Opin. Psychiatry 32, 16–21. 10.1097/YCO.0000000000000467 30394903

[B6] Carhart-Harris R. Friston K. (2010). The default-mode, ego-functions and free-energy: a neurobiological account of Freudian ideas. Brain 133, 1265–1283. 10.1093/brain/awq010 20194141 PMC2850580

[B7] Carhart-Harris R. Friston K. (2019). REBUS and the anarchic brain: toward a unified model of the brain action of psychedelics. Pharmacol. Rev. 71, 316–344. 10.1124/pr.118.017160 31221820 PMC6588209

[B8] Carhart-Harris R. Leech R. Hellyer P. J. Shanahan M. Feilding A. Tagliazucchi E.. (2014). The entropic brain: a theory of conscious states informed by neuroimaging research with psychedelic drugs. Front. Hum. Neurosci. 20. 10.3389/fnhum.2014.00020 24550805 PMC3909994

[B9] Clark A. (2012). Dreaming the whole cat: generative models, predictive processing, and the enactivist conception of perceptual experience. Mind 121, 753–771. 10.1093/mind/fzs106

[B10] Clark A. (2016). Surfing Uncertainty: Prediction, Action, and the Embodied Mind. Oxford: Oxford University Press. 10.1093/acprof:oso/9780190217013.001.0001

[B11] Crick F. Mitchison G. (1983). The function of dream sleep. Nature 304, 111–114. 10.1038/304111a0 6866101

[B12] Cutsuridis V. Yoshida M. (2017). Memory processes in medial temporal lobe: experimental, theoretical and computational approaches. Front. Syst. Neurosci. 19. 10.3389/fnsys.2017.00019 28428747 PMC5382157

[B13] Domhoff G. W. (2011). The neural substrate for dreaming: is it a subsystem of the default network? Conscious. Cogn. 20, 1163–1174. 10.1016/j.concog.2011.03.001 21450492

[B14] Dresler M. Eibl L. Fischer C. F. Wehrle R. Spoormaker V. I. Steiger, A.. (2014). Volitional components of consciousness vary across wakefulness, dreaming and lucid dreaming. Front. Psychol. 4, 987. 10.3389/fpsyg.2013.00987 24427149 PMC3877766

[B15] Edwards C. Ruby P. Malinowski J. Bennett P. Blagrove M. (2013). Dreaming and insight. Front. Psychol. 4, 979. 10.3389/fpsyg.2013.00979 24550849 PMC3872037

[B16] Fischmann T. Ambresin G. Leuzinger-Bohleber M. (2021). Dreams and Trauma Changes in the manifest dreams in psychoanalytic treatments-a psychoanalytic outcome measure. Front. Psychol. 12, 678440. 10.3389/fpsyg.2021.678440 34594260 PMC8476791

[B17] Fischmann T. Leuzinger-Bohleber M. (2017). Veränderungen von Träumen als Indikatoren für Therapieerfolg. Trauma—Zeitschrift Für Psychotraumatologie Und Ihre Anwendungen 15, 80–89.

[B18] Fischmann T. Russ M. O. Leuzinger-Bohleber M. (2013). Trauma, dream, and psychic change in psychoanalyses: a dialog between psychoanalysis and the neurosciences. Front. Hum. Neurosci. 7, 877. 10.3389/fnhum.2013.00877 24381554 PMC3865430

[B19] Fox K. C. Nijeboer S. Solomonova E. Domhoff G. W. Christoff K. (2013). Dreaming as mind wandering: evidence from functional neuroimaging and first-person content reports. Front. Hum. Neurosci. 7, 412. 10.3389/fnhum.2013.00412 23908622 PMC3726865

[B20] Freud S. (1894). The Neuro-Psychoses of Defense. Standard Edition, Vol. 3. London: Hogarth Press. p. 45–61.32811967

[B21] Freud S. (1900). “The interpretation of dreams Sigmund Freud,” in J. Strachey, J. Strachey, ed (Avon: New York).11615418

[B22] Freud S. (1915). “Instincts and their vicissitudes,” in The Standard Edition of the Complete Psychological Works of Sigmund Freud, Volume XIV (1914–1916): On the History of the Psycho-Analytic Movement, Papers on Metapsychology and Other Works. (p. 109–140).

[B23] Freud S. (1920). “Beyond the pleasure principle,” in The Standard Edition of the Complete Psychological Works of Sigmund Freud, Volume XVIII (1920–1922): Beyond the Pleasure Principle, Group Psychology and Other Works. pp. 1–64. 10.1037/11189-000

[B24] Freud S. (1923). The Ego and the Id. London: Hogarth Press and Institute of Psycho-Analysis.

[B25] Freud S. (1940). An outline of Psychoanalysis. New York: Penguin

[B26] Friston K. (2012). Prediction, perception and agency. Int. J. Psychophysiol. 83, 248–252. 10.1016/j.ijpsycho.2011.11.014 22178504 PMC3314993

[B27] Griffiths R. R. Richards W. A. McCann U. Jesse R. (2006). Psilocybin can occasion mystical-type experiences having substantial and sustained personal meaning and spiritual significance. Psychopharmacology, 187, 268–283. 10.1007/s00213-006-0457-5 16826400

[B28] Hobson J. A. (2014). Consciousness, dreams, and inference: the Cartesian theater revisited. J. Consciousness Stud. 21, 6–32.

[B29] Hobson J. A. Friston K. J. (2012). Waking and dreaming consciousness: neurobiological and functional considerations. Prog. Neurobiol. 98, 82–98. 10.1016/j.pneurobio.2012.05.003 22609044 PMC3389346

[B30] Hohwy J. (2013). The Predictive Mind. Oxford: OUP. 10.1093/acprof:oso/9780199682737.001.0001

[B31] Holze F. Vizeli P. Ley L. Müller F. Dolder P. Stocker, M.. (2021). Acute dose-dependent effects of lysergic acid diethylamide in a double-blind placebo-controlled study in healthy subjects. Neuropsychopharmacology 46, 537–544. 10.1038/s41386-020-00883-6 33059356 PMC8027607

[B32] Koslowski M. Johnson M. W. Gründer G. Betzler F. (2021). Novel treatment approaches for substance use disorders: therapeutic use of psychedelics and the role of psychotherapy. Current Addict. Rep. 9, 1–11. 10.1007/s40429-021-00401-8

[B33] Kraehenmann R. (2017). Dreams and psychedelics: neurophenomenological comparison and therapeutic implications. Curr. Neuropharmacol. 15, 1032–1042. 10.2174/1573413713666170619092629 28625125 PMC5652011

[B34] Kraehenmann R. Pokorny D. Aicher H. Preller K. H. Pokorny T. Bosch O.. (2017). LSD increases primary process thinking *via* serotonin 2A receptor activation. Front. Pharmacol. 8, 814. 10.3389/fphar.2017.00814 29167644 PMC5682333

[B35] Letheby C. Gerrans P. (2017). Self unbound: ego dissolution in psychedelic experience. Neurosci. Consciousness 3. 10.1093/nc/nix016 30042848 PMC6007152

[B36] Loo M.-R. Cheng S.-K. (2022). Dream lucidity positively correlates with reality monitoring. Conscious. Cogn. 105, 103414. 10.1016/j.concog.2022.103414 36183604

[B37] Michaiel A. M. Parker P. R. Niell C. M. (2019). A hallucinogenic serotonin-2A receptor agonist reduces visual response gain and alters temporal dynamics in mouse V1. Cell Rep. 26, 3475–3483.e3474. 10.1016/j.celrep.2019.02.104 30917304 PMC6559379

[B38] Millière R. (2017). Looking for the self: phenomenology, neurophysiology and philosophical significance of drug-induced ego dissolution. Front. Hum. Neurosci. 11, 245. 10.3389/fnhum.2017.00245 28588463 PMC5441112

[B39] Millière R. Carhart-Harris R. L. Roseman L. Trautwein F-. M. Berkovich-Ohana A. (2018). Psychedelics, meditation, and self-consciousness. Front. Psychol. 9, 1475. 10.3389/fpsyg.2018.01475 30245648 PMC6137697

[B40] Moser U. Hortig V. (2019). Mikrowelt Traum—Affektregulierung und Reflexion. Frankfurt a. M.: Brandes and Apsel.

[B41] Moser U. von Zeppelin I. (1996). Der geträumte Traum: wie Träume entstehen und sich verändern: Kohlhammer.

[B42] Mota-Rolim S. A. (2020). On moving the eyes to flag lucid dreaming. Front. Neurosci. 14, 361. 10.3389/fnins.2020.00361 32351360 PMC7174658

[B43] Palhano-Fontes F. Mota-Rolim S. Lobão-Soares B. Galvão-Coelho N. Maia-Oliveira J. P. Araújo D. B.. (2021). “Recent evidence on the antidepressant effects of ayahuasca,” in Ayahuasca Healing and Science (Berlin: Springer). p. 21–41. 10.1007/978-3-030-55688-4_2

[B44] Panksepp J. Wilson C. G. (2016). “Brain SEEKING circuitry in neuroeconomics: a unifying hypothesis for the role of dopamine-energized arousal of the medial forebrain bundle in enthusiasm-guiding decision-making,” in Neuroeconomics (Berlin: Springer). p. 231–252. 10.1007/978-3-642-35923-1_13

[B45] Preller K. H. Vollenweider F. X. (2018). Phenomenology, structure, and dynamic of psychedelic states. Curr. Top. Behav. Neurosci. 36, 221–256. 10.1007/7854_2016_459 28025814

[B46] Revonsuo A. (2000). The reinterpretation of dreams: an evolutionary hypothesis of the function of dreaming. Behav. Brain Sci. 23, 877–901. 10.1017/S0140525X00004015 11515147

[B47] Robbins M. (2018). The primary process: Freud's profound yet neglected contribution to the psychology of consciousness. Psychoanalytic Inquiry 38, 186–197. 10.1080/07351690.2018.1430965

[B48] Ruby P. M. (2011). Experimental research on dreaming: state of the art and neuropsychoanalytic perspectives. Front. Psychol. 2, 286. 10.3389/fpsyg.2011.00286 22121353 PMC3220269

[B49] Sanz C. Zamberlan F. Erowid E. Erowid F. Tagliazucchi E. (2018). The experience elicited by hallucinogens presents the highest similarity to dreaming within a large database of psychoactive substance reports. Front. Neurosci. 12, 7. 10.3389/fnins.2018.00007 29403350 PMC5786560

[B50] Scarpelli S. Nadorff M. R. Bjorvatn B. Chung F. Dauvilliers Y. Espie C.. (2022). Nightmares in people with COVID-19: did Coronavirus infect our dreams? Nat. Sci. Sleep 14, 93. 10.2147/NSS.S344299 35115852 PMC8800372

[B51] Schmidt T. T. Berkemeyer H. (2018). The altered states database: psychometric data of altered states of consciousness. Front. Psychol. 9, 1028. 10.3389/fpsyg.2018.01028 30013493 PMC6036510

[B52] Schultes R. E. Hofmann A. (1979). Plants of the Gods: Origins of Hallucinogenic Use. New York: McGraw-Hill.

[B53] Solms M. (2000). Dreaming and REM sleep are controlled by different brain mechanisms. Behav. Brain Sci. 23, 843–850. 10.1017/S0140525X00003988 11515144

[B54] Solms M. (2013). The conscious id. Neuropsychoanalysis 15, 5–19. 10.1080/15294145.2013.10773711

[B55] Solms M. (2014). The Neuropsychology of Dreams: A Clinico-Anatomical Study. Milton Park: Psychology Press. 10.4324/9781315806440

[B56] Solms M. (2018). The neurobiological underpinnings of psychoanalytic theory and therapy. Front. Behav. Neurosci. 12, 294. 10.3389/fnbeh.2018.00294 30564106 PMC6288296

[B57] Solms M. (2019). The hard problem of consciousness and the free energy principle. Front. Psychol. 9, 2714. 10.3389/fpsyg.2018.02714 30761057 PMC6363942

[B58] Solms M. (2021). The Hidden Spring: A Journey to the Source of Consciousness. New York: WW Norton and Company. 10.53765/20512201.28.11.153

[B59] Spanò G. Pizzamiglio G. McCormick C. Clark I. A. De Felice S. Miller T. D.. (2020). Dreaming with hippocampal damage. Elife 9, e56211. 10.7554/eLife.56211.sa2 32508305 PMC7279885

[B60] Spiegelhalter D. Rice K. (2009). Bayesian statistics. Scholarpedia 4, 5230. 10.4249/scholarpedia.5230

[B61] Stigler M. Pokorny D. (2001). Emotions and primary process in guided imagery psychotherapy: computerized text-analytic measures. Psychother. Res. 11, 415–431. 10.1093/ptr/11.4.415

[B62] Studerus E. Kometer M. Hasler F. Vollenweider F. X. (2011). Acute, subacute and long-term subjective effects of psilocybin in healthy humans: a pooled analysis of experimental studies. J. Psychopharmacol. 25, 1434–1452. 10.1177/0269881110382466 20855349

[B63] Sui J. Humphreys G. W. (2015). The integrative self: how self-reference integrates perception and memory. Trends Cogn. Sci. 19, 719–728. 10.1016/j.tics.2015.08.015 26447060

[B64] Tuominen J. Stenberg T. Revonsuo A. Valli K. (2019). Social contents in dreams: an empirical test of the social simulation theory. Conscious. Cogn. 69, 133–145. 10.1016/j.concog.2019.01.017 30769273

[B65] Vollenweider F. X. Kometer M. (2010). The neurobiology of psychedelic drugs: implications for the treatment of mood disorders. Nat. Rev. Neurosci. 11, 642–651. 10.1038/nrn2884 20717121

[B66] Windt J. M. (2015). Dreaming: A Conceptual Framework for Philosophy of Mind and Empirical Research. Cambridge: MIT press. 10.7551/mitpress/9780262028677.001.0001

[B67] Wittmann L. Anstadt T. Fischmann T. Hau S. Kempe S. Herot K.. (2017). Ein Traum, zwei Methoden. J. Psychoanalyze 58, 99–129. 10.18754/jfp.58.6

[B68] Wolff M. Evens R. Mertens L. J. Koslowski M. Betzler F. Grunder G.. (2020). Learning to let go: a cognitive-behavioral model of how psychedelic therapy promotes acceptance. Front Psychiatry 11, 5. 10.3389/fpsyt.2020.00005 32153433 PMC7046795

[B69] Yaden D. B. Earp D. Graziosi M. Friedman-Wheeler D. Luoma J. B. Johnson M. W.. (2022). Psychedelics and psychotherapy: cognitive-behavioral approaches as default. Front. Psychol. 13, 1604. 10.3389/fpsyg.2022.873279 35677124 PMC9169963

